# Supplementary material for: Holothuriophilus trapeziformis Nauck, 1880 (Decapoda: Pinnotheridae) from the Pacific coast of Mexico: taxonomic revision based on integrative taxonomy
Source: PeerJ. 2022 Feb 23;10:e12774. doi: 10.7717/peerj.12774 (PMC8881913; doi:10.7717/peerj.12774)
Supplement: Supplemental Information 1 — CW, carapace width in millimeters; CL, carapace length in millimeters; UMAR-CNC, Catalog number in the scientific collection OAX-CC-249-11; NU not used for COI sequence in BOLD; BOLD-ID, specimen identifier in the Barcode of Life Data System; BOLD-ID in bold letter, indicates BIN (BOLD:ADE9974) association. Locality arrangement goes from north to south. [file peerj-10-12774-s001.docx]

**Supplemental Table S1. Specimens of *Holothuriophilus trapeziformis* from the Pacific coast of México analyzed for the present study.** CW/CL, carapace width and length, respectively, in millimeters; UMAR-CNC, Catalog number in the scientific collection OAX-CC-249-11; NU not used for COI sequence in BOLD; BOLD-ID, specimen identifier in the Barcode of Life Data System. Locality arrangement goes from north to south.

| UMAR-CNC | BOLD-ID | Sex | CW/CL | Date of collection | Locality | Latitud (N) | Longitud (W) | Deep (meters) | Collector |
| --- | --- | --- | --- | --- | --- | --- | --- | --- | --- |
| DECA-1187 | CH736-A1 | male | 9.1/6.1 | Sep. 09, 2017 | playa Pinitos, Sinaloa | 23°12'30.71'' | 106°25'40.78'' | 1 | FCC |
| DECA-1188 | CH736-A2 | female | 9/6 | Sep. 09, 2017 | playa Pinitos, Sinaloa | 23°12'30.71'' | 106°25'40.78'' | 1 | FCC |
| DECA-1189 | CH736-A3 | female | 9/5.8 | Sep. 09, 2017 | playa Pinitos, Sinaloa | 23°12'30.71'' | 106°25'40.78'' | 1 | FCC |
| DECA-1190 | CH736-A4 | male | 8/5 | Sep. 09, 2017 | playa Pinitos, Sinaloa | 23°12'30.71'' | 106°25'40.78'' | 1 | FCC |
| DECA-1191 | CH736-A5 | male | 7.6/5 | Sep. 09, 2017 | playa Pinitos, Sinaloa | 23°12'30.71'' | 106°25'40.78'' | 1 | FCC |
| DECA-1192 | CH736-A6 | female | 8/5 | Sep. 09, 2017 | playa Pinitos, Sinaloa | 23°12'30.71'' | 106°25'40.78'' | 1 | FCC |
| DECA-1193 | CH736-A7 | female | 9.1/6 | Sep. 09, 2017 | playa Pinitos, Sinaloa | 23°12'30.71'' | 106°25'40.78'' | 1 | FCC |
| DECA-1269 | NU | female | 9/6 | Sep. 09, 2017 | playa Pinitos, Sinaloa | 23°12'30.71'' | 106°25'40.78'' | 1 | FCC |
| DECA-1143 | CH736-A12 | ovigerous female | 10.5/6 | Nov. 30, 2010 | playa Nudista, Guerrero | 17°40'31.26'' | 101°39'3.06'' | 1.5 | RGF |
| DECA-1144 | CH736-B12 | female | 7.9/4 | Dec. 02, 2010 | playa Zacatoso, Guerrero | 17°39'15.12'' | 101°37'18.66'' | 5-6 | FCC |
| DECA-1145 | CH736-B9 | ovigerous female | 10.9/6.5 | Dec. 02, 2010 | playa Zacatoso, Guerrero | 17°39'15.12'' | 101°37'18.66'' | 5-6 | FCC |
| DECA-1146 | CH736-B10 | ovigerous female | 10/6 | Dec. 02, 2010 | playa Zacatoso, Guerrero | 17°39'15.12'' | 101°37'18.66'' | 5-6 | FCC |
| DECA-1147 | CH736-B11 | ovigerous female | 7.3/5 | Dec. 02, 2010 | playa Zacatoso, Guerrero | 17°39'15.12'' | 101°37'18.66'' | 5-6 | FCC |
| DECA-1148 | CH736-B8 | male | 8/5.5 | Dec. 02, 2010 | playa Zacatoso, Guerrero | 17°39'15.12'' | 101°37'18.66'' | 5-6 | FCC |
| DECA-1149 | CH736-B1 | ovigerous female | 8/5 | Dec. 02, 2010 | playa Caleta de Chón, Guerreo | 17°36'55.68'' | 101°33'17.64'' | 3-6 | FCC |
| DECA-1150 | CH736-B2 | ovigerous female | 9/6 | Dec. 02, 2010 | playa Caleta de Chón, Guerreo | 17°36'55.68'' | 101°33'17.64'' | 3-6 | FCC |
| DECA-1151 | CH736-B3 | ovigerous female | 8/5 | Dec. 02, 2010 | playa Caleta de Chón, Guerreo | 17°36'55.68'' | 101°33'17.64'' | 3-6 | FCC |
| DECA-1152 | CH736-B4 | female | 5.1/3 | Dec. 02, 2010 | playa Caleta de Chón, Guerreo | 17°39'15.12'' | 101°37'18.66'' | 3-6 | FCC |
| DECA-1153 | CH736-B5 | female | 8.2/6 | Dec. 02, 2010 | playa Caleta de Chón, Guerreo | 17°36'55.68'' | 101°33'17.64'' | 3-6 | FCC |
| DECA-1154 | CH736-B6 | male | 5.5/3.2 | Dec. 02, 2010 | playa Caleta de Chón, Guerreo | 17°39'15.12'' | 101°37'18.66'' | 3-6 | FCC |
| DECA-1155 | CH736-B7 | female | 7/5 | Dec. 02, 2010 | playa Caleta de Chón, Guerreo | 17°36'55.68'' | 101°33'17.64'' | 3-6 | FCC |
| DECA-1161 | CH736-A10 | ovigerous female | 12/7 | Aug. 12, 2009 | playa Coral, Oaxaca | 15°51'30.81" | 97° 3'56.27" | 3 | CCA & KMB |
| DECA-307 | CH736-A08 | female | 10.5/7 | Nov. 28, 2008 | playa Agua Blanca, Oaxaca | 15°43'54.34" | 96°48'46.59" | 5 | AEV & AGF |
| DECA-1156 | CH736-A11 | ovigerous female | 12/7.9 | Oct. 09, 2010 | playa Agua Blanca, Oaxaca | 15°43'58.45'' | 96°48'50.66'' | 1.5 | FCC |
| DECA-1157 | CH670-A1 | female | 9.5/7 | May. 03, 2016 | playa Agua Blanca, Oaxaca | 15°43'58.45'' | 96°48'50.66'' | 1 | FCC |
| DECA-1158 | CH670-A02 | female | 10/6.6 | May. 03, 2016 | playa Agua Blanca, Oaxaca | 15°43'58.45'' | 96°48'50.66'' | 1 | FCC |
| DECA-1159 | CH670-A03 | ovigerous female | 10/6.4 | May. 03, 2016 | playa Agua Blanca, Oaxaca | 15°43'58.45'' | 96°48'50.66'' | 1 | FCC |
| DECA-1160 | CH670-A04 | ovigerous female | 12/8 | May. 03, 2016 | playa Agua Blanca, Oaxaca | 15°43'58.45'' | 96°48'50.66'' | 1 | FCC |
| DECA-1162 | CH736-G5 | female | 10.5/6.5 | March 3, 2017 | playa Camarón, Oaxaca | 15°39'45.48'' | 96°31'32.49'' | 1 | KLFL |
| DECA-1163 | CH736-G6 | ovigerous female | 13/8 | March 3, 2017 | playa Camarón, Oaxaca | 15°39'45.48'' | 96°31'32.49'' | 1 | KLFL |
| DECA-1164 | NU | female | 10/6 | March 3, 2017 | playa Camarón, Oaxaca | 15°39'45.48'' | 96°31'32.49'' | 1 | KLFL |
| DECA-308 | CH736-A9 | male | 11/7 | Dec. 05, 2009 | playa Panteón, Oaxaca | 15°39'50.91'' | 96°29'40.26'' | 10 | AHM & HMC |
| DECA-1270 | NU | male | 8/6 | March 02, 2018 | playa Estacahuite, Oaxaca | 15°40'8.52'' | 96°28'53.48'' | 4 | KLFL & FBV |
| DECA-1165 | CH736-H3 | ovigerous female | 9.6/6 | March 17, 2017 | playa Estacahuite, Oaxaca | 15°40'8.52'' | 96°28'53.48'' | 1 | KLFL |
| DECA-1166 | CH736-H4 | ovigerous female | 10/6.5 | May. 12, 2017 | playa Estacahuite, Oaxaca | 15°40'8.52'' | 96°28'53.48'' | 1.5 | KLFL & FBV |
| DECA-1167 | CH736-H5 | ovigerous female | 10.5/7 | May. 12, 2017 | playa Estacahuite, Oaxaca | 15°40'8.52'' | 96°28'53.48'' | 1.5 | KLFL & FBV |
| DECA-1168 | CH736-H6 | female | 7.8/5.2 | May. 12, 2017 | playa Estacahuite, Oaxaca | 15°40'8.52'' | 96°28'53.48'' | 1.5 | KLFL & FBV |
| DECA-1169 | CH736-H7 | female | 9.5/6.5 | May. 12, 2017 | playa Estacahuite, Oaxaca | 15°40'8.52'' | 96°28'53.48'' | 1.5 | KLFL & FBV |
| DECA-1170 | CH736-H8 | ovigerous female | 10.8/6.2 | May. 12, 2017 | playa Estacahuite, Oaxaca | 15°40'8.52'' | 96°28'53.48'' | 1.5 | KLFL & FBV |
| DECA-1171 | CH736-H9 | ovigerous female | 11/7.5 | May. 12, 2017 | playa Estacahuite, Oaxaca | 15°40'8.52'' | 96°28'53.48'' | 1.5 | KLFL & FBV |
| DECA-1172 | CH736-H10 | ovigerous female | 9/6 | May. 12, 2017 | playa Estacahuite, Oaxaca | 15°40'8.52'' | 96°28'53.48'' | 1.5 | KLFL & FBV |
| DECA-1173 | CH736-H11 | female | 5.5/3.5 | May. 12, 2017 | playa Estacahuite, Oaxaca | 15°40'8.52'' | 96°28'53.48'' | 1.5 | KLFL & FBV |
| DECA-1174 | NU | male | 9/6 | March 26, 2015 | playa Estacahuite, Oaxaca | 15°40'8.52'' | 96°28'53.48'' | 5-6 | VCH |
| DECA-1175 | CH708-H8 | female | 8/5 | Aug. 02, 2017 | playa La Tijera, Oaxaca | 15°41'14'' | 96°26'35.51' | 1 | FCC |
| DECA-1176 | CH736-G7 | female | 10.5/6 | Apr. 07, 2017 | bahía San Agustín, Oaxaca | 15°41'22.56'' | 96°14'11.7'' | 3 | KLFL & JDGV |
| DECA-1177 | CH736-G11 | female | 11/7 | Apr. 07, 2017 | bahía San Agustín, Oaxaca | 15°41'22.56'' | 96°14'11.7'' | 3 | KLFL & JDGV |
| DECA-1178 | CH736-H2 | female | 9.5/6 | Apr. 07, 2017 | bahía San Agustín, Oaxaca | 15°41'22.56'' | 96°14'11.7'' | 3 | KLFL & JDGV |
| DECA-1179 | CH736-G8 | ovigerous female | 11/7 | Apr. 07, 2017 | bahía San Agustín, Oaxaca | 15°41'22.56'' | 96°14'11.7'' | 3 | KLFL & JDGV |
| DECA-1180 | CH736-G9 | ovigerous female | 10/6.5 | Apr. 07, 2017 | bahía San Agustín, Oaxaca | 15°41'22.56'' | 96°14'11.7'' | 3 | KLFL & JDGV |
| DECA-1181 | CH736-G10 | ovigerous female | 10.8/7 | Apr. 07, 2017 | bahía San Agustín, Oaxaca | 15°41'22.56'' | 96°14'11.7'' | 3 | KLFL & JDGV |
| DECA-1182 | CH736-H1 | ovigerous female | 8/5 | Apr. 07, 2017 | bahía San Agustín, Oaxaca | 15°41'22.56'' | 96°14'11.7'' | 3 | KLFL & JDGV |
| DECA-1183 | CH736-G12 | male | 9.5/6.5 | Apr. 07, 2017 | bahía San Agustín, Oaxaca | 15°41'22.56'' | 96°14'11.7'' | 3 | KLFL & JDGV |
| DECA-1184 | CH670-A05 | ovigerous female | 8.1/5.1 | May. 04, 2017 | playa El Tejón, Oaxaca | 15°45'46.00'' | 96°6'42.63'' | 3 | FCC |
| DECA-1185 | CH670-A06 | ovigerous female | 10.1/6.5 | May. 04, 2017 | playa El Tejón, Oaxaca | 15°45'46.00'' | 96°6'42.63'' | 3 | FCC |
| DECA-1186 | CH670-A07 | ovigerous female | 10/6.5 | May. 04, 2017 | playa El Tejón, Oaxaca | 15°45'46.00'' | 96°6'42.63'' | 3 | FCC |
