# Supplementary material for: Holothuriophilus trapeziformis Nauck, 1880 (Decapoda: Pinnotheridae) from the Pacific coast of Mexico: taxonomic revision based on integrative taxonomy
Source: PeerJ. 2022 Feb 23;10:e12774. doi: 10.7717/peerj.12774 (PMC8881913; doi:10.7717/peerj.12774)
Supplement: Supplemental Information 2 [file peerj-10-12774-s002.docx]

**Supplemental Table S2.** **Public sequences used for maximum likelihood analyses. An “*” indicates that the specimen and the sequence were obtained by us.**

| Identification | BOLD-ID | BOLD-BIN | BOLD sequence page | GenBank access number | Exact Site |
| --- | --- | --- | --- | --- | --- |
| **Austinixa* sp. | CH736-F7 | BOLD:ADY5222 | PINMX223-18 | MW544436 | Rancho La Encrucijada, Chiapas, Mexico |
| **Austinixa* sp. | CH736-F6 | BOLD:ADY5222 | PINMX222-18 | MW544387 | Rancho La Encrucijada, Chiapas, Mexico |
| **Austinixa* sp. | CH736-F5 | BOLD:ADY5222 | PINMX221-18 | MW544353 | Rancho La Encrucijada, Chiapas, Mexico |
| **Austinixa* sp. | CH736-F4 | BOLD:ADY5222 | PINMX220-18 | MW544372 | Rancho La Encrucijada, Chiapas, Mexico |
| **Austinixa* sp. | CH736-F3 | BOLD:ADY5222 | PINMX219-18 | MW544421 | Rancho La Encrucijada, Chiapas, Mexico |
| **Austinixa* sp. | CH736-F2 | BOLD:ADY5222 | PINMX218-18 | MW544340 | Rancho La Encrucijada, Chiapas, Mexico |
| *Austinixa felipensis* | AF501574 | ND | GBCMD0154-06 | AF501574 | Mexico |
| *Austinotheres angelicus* | KU172684 | ND | GBCMD21091-19 | KU172684 | Panama |
| **Calyptraeotheres granti* | CH736-D11 | BOLD:ADY2430 | PINMX203-18 | MW544437 | San Agustin Bay, Mexico |
| **Calyptraeotheres* sp. | CH670-C09 | BOLD:ADF0478 | PINMX033-16 | MW544365 | San Agustin Bay, Oaxaca, Mexico |
| **Calyptraeotheres* sp. | CH736-C1 | BOLD:ADY0082 | PINMX181-18 | MW544422 | Morro de La India, Oaxaca, Mexico |
| **Calyptraeotheres* sp. | CH736-C3 | BOLD:ADY2430 | PINMX183-18 | MW544400 | Morro de La India, Oaxaca, Mexico |
| **Calyptraeotheres* sp. | CH736-C5 | BOLD:ADY2430 | PINMX185-18 | MW544415 | Morro de La India, Oaxaca, Mexico |
| **Calyptraeotheres* sp. | CH736-C7 | BOLD:ADY2430 | PINMX187-18 | MW544325 | Morro de La India, Oaxaca, Mexico |
| **Calyptraeotheres* sp. | CH736-C11 | BOLD:ADY0082 | PINMX191-18 | MW544373 | La Blanca, Oaxaca, Mexico |
| **Calyptraeotheres* sp. | CH736-D9 | BOLD:ADY2430 | PINMX201-18 | MW544316 | Morro del Cerro Colorado, Guerrero, Mexico |
| **Calyptraeotheres* sp. | CH736-E5 | BOLD:ADY2430 | PINMX209-18 | MW544321 | dock of Principal beach, Puerto Angel, Mexico |
| *Calyptraeotheres politus* | JX839511 | BOLD:ABV8132 | GBCMA7000-14 | JX839511 | Chile |
| *Calyptraeotheres politus* | JX839512 | BOLD:ABV8132 | GBCMA7001-14 | JX839512 | Chile |
| *Calyptraeotheres politus* | JX839513 | BOLD:ABV8132 | GBCMA7002-14 | JX839513 | Chile |
| *Calyptraeotheres politus* | JX839514 | BOLD:ABV8132 | GBCMA7003-14 | JX839514 | Chile |
| *Calyptraeotheres politus* | JX839515 | BOLD:ABV8132 | GBCMA7004-14 | JX839515 | Chile |
| *Calyptraeotheres politus* | JX839516 | BOLD:ABV8132 | GBCMA7005-14 | JX839516 | Chile |
| *Calyptraeotheres politus* | JX839517 | BOLD:ABV8132 | GBCMA7006-14 | JX839517 | Chile |
| *Calyptraeotheres politus* | JX839518 | BOLD:ABV8132 | GBCMA7007-14 | JX839518 | Chile |
| *Calyptraeotheres politus* | JX839519 | BOLD:ABV8132 | GBCMA7008-14 | JX839519 | Chile |
| *Calyptraeotheres politus* | JX839520 | BOLD:ABV8132 | GBCMA7009-14 | JX839520 | Chile |
| **Dissodactylus schmitti* | CH670-A08 | BOLD:ADE2628 | PINMX008-16 | MW544396 | El Violin Bay, Oaxaca Mexico |
| **Dissodactylus schmitti* | CH670-A09 | BOLD:ADE2628 | PINMX009-16 | MW544352 | El Violin Bay, Oaxaca Mexico |
| **Dissodactylus schmitti* | CH670-A10 | BOLD:ADE2628 | PINMX010-16 | MW544420 | El Violin Bay, Oaxaca Mexico |
| **Dissodactylus schmitti* | CH670-A11 | BOLD:ADE2628 | PINMX011-16 | MW544334 | El Violin Bay, Oaxaca Mexico |
| **Dissodactylus schmitti* | CH670-B03 | BOLD:ADE2628 | PINMX015-16 | MW544401 | El Violin Bay, Oaxaca Mexico |
| **Dissodactylus schmitti* | CH670-B04 | BOLD:ADE2628 | PINMX016-16 | MW544319 | El Violin Bay, Oaxaca Mexico |
| **Dissodactylus schmitti* | CH670-B05 | BOLD:ADE2628 | PINMX017-16 | MW544346 | El Violin Bay, Oaxaca Mexico |
| **Dissodactylus schmitti* | CH670-B07 | BOLD:ADE2628 | PINMX019-16 | MW544416 | El Violin Bay, Oaxaca Mexico |
| **Dissodactylus schmitti* | CH670-B08 | BOLD:ADE2628 | PINMX020-16 | MW544302 | El Violin Bay, Oaxaca Mexico |
| **Dissodactylus schmitti* | CH670-B09 | BOLD:ADE2628 | PINMX021-16 | MW544294 | El Violin Bay, Oaxaca Mexico |
| **Dissodactylus schmitti* | CH670-B10 | BOLD:ADE2628 | PINMX022-16 | MW544347 | El Violin Bay, Oaxaca Mexico |
| **Dissodactylus schmitti* | CH670-B11 | BOLD:ADE2628 | PINMX023-16 | MW544298 | El Violin Bay, Oaxaca Mexico |
| **Dissodactylus schmitti* | CH670-B12 | BOLD:ADE2628 | PINMX024-16 | MW544314 | El Violin Bay, Oaxaca Mexico |
| **Dissodactylus schmitti* | CH670-C02 | BOLD:ADE2628 | PINMX026-16 | MW544377 | El Violin Bay, Oaxaca Mexico |
| **Dissodactylus schmitti* | CH670-C03 | BOLD:ADE2628 | PINMX027-16 | MW544308 | El Violin Bay, Oaxaca Mexico |
| **Dissodactylus schmitti* | CH670-C04 | BOLD:ADE2628 | PINMX028-16 | MW544335 | El Violin Bay, Oaxaca Mexico |
| **Dissodactylus schmitti* | CH670-C05 | BOLD:ADE2628 | PINMX029-16 | MW544341 | El Violin Bay, Oaxaca Mexico |
| **Dissodactylus schmitti* | CH670-C06 | BOLD:ADE2628 | PINMX030-16 | MW544397 | El Violin Bay, Oaxaca Mexico |
| **Dissodactylus schmitti* | CH670-C07 | BOLD:ADE2628 | PINMX031-16 | MW544339 | El Violin Bay, Oaxaca Mexico |
| **Dissodactylus schmitti* | CH670-C08 | BOLD:ADE2628 | PINMX032-16 | MW544423 | El Violin Bay, Oaxaca Mexico |
| **Dissodactylus schmitti* | CH708-D02 | BOLD:ADE2628 | PINMX098-18 | MW544310 | Chahue beach, Oaxaca, Mexico |
| **Dissodactylus schmitti* | CH708-D03 | BOLD:ADE2628 | PINMX099-18 | MW544350 | Chahue beach, Oaxaca, Mexico |
| **Dissodactylus schmitti* | CH708-D04 | BOLD:ADE2628 | PINMX100-18 | MW544432 | Chahue beach, Oaxaca, Mexico |
| **Dissodactylus schmitti* | CH708-D05 | BOLD:ADE2628 | PINMX101-18 | MW544371 | Chahue beach, Oaxaca, Mexico |
| **Dissodactylus schmitti* | CH708-D06 | BOLD:ADE2628 | PINMX102-18 | MW544410 | Chahue beach, Oaxaca, Mexico |
| **Dissodactylus schmitti* | CH708-D07 | BOLD:ADE2628 | PINMX103-18 | MW544326 | Chahue beach, Oaxaca, Mexico |
| **Dissodactylus schmitti* | CH708-D08 | BOLD:ADE2628 | PINMX104-18 | MW544311 | Chahue beach, Oaxaca, Mexico |
| **Dissodactylus schmitti* | CH709-D09 | BOLD:ADE2628 | PINMX105-18 | MW544343 | Chahue beach, Oaxaca, Mexico |
| **Dissodactylus schmitti* | CH708-D10 | BOLD:ADE2628 | PINMX106-18 | MW544309 | Chahue beach, Oaxaca, Mexico |
| **Dissodactylus schmitti* | CH708-D11 | BOLD:ADE2628 | PINMX107-18 | MW544342 | Chahue beach, Oaxaca, Mexico |
| **Dissodactylus schmitti* | CH708-D12 | BOLD:ADE2628 | PINMX108-18 | MW544405 | Chahue beach, Oaxaca, Mexico |
| **Dissodactylus schmitti* | CH708-E01 | BOLD:ADE2628 | PINMX109-18 | MW544336 | Chahue beach, Oaxaca, Mexico |
| **Dissodactylus schmitti* | CH708-E02 | BOLD:ADE2628 | PINMX110-18 | MW544297 | Chahue beach, Oaxaca, Mexico |
| **Dissodactylus schmitti* | CH708-E03 | BOLD:ADE2628 | PINMX111-18 | MW544360 | Chahue beach, Oaxaca, Mexico |
| **Dissodactylus schmitti* | CH708-E04 | BOLD:ADE2628 | PINMX112-18 | MW544382 | Chahue beach, Oaxaca, Mexico |
| **Dissodactylus schmitti* | CH708-E05 | BOLD:ADE2628 | PINMX113-18 | MW544433 | Chahue beach, Oaxaca, Mexico |
| **Dissodactylus schmitti* | CH708-E06 | BOLD:ADE2628 | PINMX114-18 | MW544375 | Chahue beach, Oaxaca, Mexico |
| **Dissodactylus schmitti* | CH708-E07 | BOLD:ADE2628 | PINMX115-18 | MW544443 | Chahue beach, Oaxaca, Mexico |
| **Dissodactylus schmitti* | CH708-E08 | BOLD:ADE2628 | PINMX116-18 | MW544329 | Chahue beach, Oaxaca, Mexico |
| **Dissodactylus schmitti* | CH708-E09 | BOLD:ADE2628 | PINMX117-18 | MW544299 | Chahue beach, Oaxaca, Mexico |
| **Dissodactylus schmitti* | CH708-E10 | BOLD:ADE2628 | PINMX118-18 | MW544381 | Chahue beach, Oaxaca, Mexico |
| **Dissodactylus schmitti* | CH708-E11 | BOLD:ADE2628 | PINMX119-18 | MW544362 | Chahue beach, Oaxaca, Mexico |
| **Dissodactylus schmitti* | CH708-E12 | BOLD:ADE2628 | PINMX120-18 | MW544359 | Chahue beach, Oaxaca, Mexico |
| **Dissodactylus schmitti* | CH708-F01 | BOLD:ADE2628 | PINMX121-18 | MW544438 | Chahue beach, Oaxaca, Mexico |
| **Dissodactylus schmitti* | CH708-F02 | BOLD:ADE2628 | PINMX122-18 | MW544324 | Chahue beach, Oaxaca, Mexico |
| **Dissodactylus schmitti* | CH708-F03 | BOLD:ADE2628 | PINMX123-18 | MW544374 | Chahue beach, Oaxaca, Mexico |
| **Dissodactylus schmitti* | CH708-F04 | BOLD:ADE2628 | PINMX124-18 | MW544305 | Chahue beach, Oaxaca, Mexico |
| **Dissodactylus schmitti* | CH708-F05 | BOLD:ADE2628 | PINMX125-18 | MW544429 | Chahue beach, Oaxaca, Mexico |
| **Dissodactylus schmitti* | CH708-F06 | BOLD:ADE2628 | PINMX126-18 | MW544351 | Chahue beach, Oaxaca, Mexico |
| **Dissodactylus schmitti* | CH708-F07 | BOLD:ADE2628 | PINMX127-18 | MW544349 | Chahue beach, Oaxaca, Mexico |
| **Dissodactylus schmitti* | CH708-F08 | BOLD:ADE2628 | PINMX128-18 | MW544337 | Chahue beach, Oaxaca, Mexico |
| **Dissodactylus schmitti* | CH708-F09 | BOLD:ADE2628 | PINMX129-18 | MW544385 | Chahue beach, Oaxaca, Mexico |
| **Dissodactylus schmitti* | CH708-F10 | BOLD:ADE2628 | PINMX130-18 | MW544318 | Chahue beach, Oaxaca, Mexico |
| **Dissodactylus schmitti* | CH708-F11 | BOLD:ADE2628 | PINMX131-18 | MW544320 | Chahue beach, Oaxaca, Mexico |
| **Dissodactylus schmitti* | CH708-F12 | BOLD:ADE2628 | PINMX132-18 | MW544403 | Chahue beach, Oaxaca, Mexico |
| **Dissodactylus schmitti* | CH708-G01 | BOLD:ADE2628 | PINMX133-18 | MW544364 | Chahue beach, Oaxaca, Mexico |
| **Dissodactylus schmitti* | CH708-G02 | BOLD:ADE2628 | PINMX134-18 | MW544398 | Chahue beach, Oaxaca, Mexico |
| **Dissodactylus schmitti* | CH708-G03 | BOLD:ADE2628 | PINMX135-18 | MW544412 | Chahue beach, Oaxaca, Mexico |
| **Dissodactylus schmitti* | CH708-G04 | BOLD:ADE2628 | PINMX136-18 | MW544317 | Chahue beach, Oaxaca, Mexico |
| **Dissodactylus schmitti* | CH708-G05 | BOLD:ADE2628 | PINMX137-18 | MW544368 | Chahue beach, Oaxaca, Mexico |
| **Dissodactylus schmitti* | CH708-G06 | BOLD:ADE2628 | PINMX138-18 | MW544430 | Chahue beach, Oaxaca, Mexico |
| **Dissodactylus schmitti* | CH708-G07 | BOLD:ADE2628 | PINMX139-18 | MW544380 | Chahue beach, Oaxaca, Mexico |
| **Dissodactylus schmitti* | CH708-G08 | BOLD:ADE2628 | PINMX140-18 | MW544354 | Chahue beach, Oaxaca, Mexico |
| **Dissodactylus schmitti* | CH708-G09 | BOLD:ADE2628 | PINMX141-18 | MW544411 | Chahue beach, Oaxaca, Mexico |
| **Dissodactylus schmitti* | CH708-G10 | BOLD:ADE2628 | PINMX142-18 | MW544332 | Chahue beach, Oaxaca, Mexico |
| **Dissodactylus schmitti* | CH708-G11 | BOLD:ADE2628 | PINMX143-18 | MW544304 | Chahue beach, Oaxaca, Mexico |
| **Dissodactylus schmitti* | CH708-G12 | BOLD:ADE2628 | PINMX144-18 | MW544344 | Chahue beach, Oaxaca, Mexico |
| **Dissodactylus schmitti* | CH708-H01 | BOLD:ADE2628 | PINMX145-18 | MW544378 | Chahue beach, Oaxaca, Mexico |
| **Dissodactylus schmitti* | CH708-H02 | BOLD:ADE2628 | PINMX146-18 | MW544300 | Chahue beach, Oaxaca, Mexico |
| **Dissodactylus schmitti* | CH708-H03 | BOLD:ADE2628 | PINMX147-18 | MW544431 | Chahue beach, Oaxaca, Mexico |
| **Dissodactylus schmitti* | CH708-H04 | BOLD:ADE2628 | PINMX148-18 | MW544333 | Chahue beach, Oaxaca, Mexico |
| **Dissodactylus schmitti* | CH708-H05 | BOLD:ADE2628 | PINMX149-18 | MW544391 | Chahue beach, Oaxaca, Mexico |
| **Dissodactylus schmitti* | CH708-H06 | BOLD:ADE2628 | PINMX150-18 | MW544327 | Chahue beach,, Oaxaca, Mexico |
| **Dissodactylus schmitti* | CH708-H07 | BOLD:ADE2628 | PINMX151-18 | MW544390 | Del Organo Bay, Oaxaca, Mexico |
| **Dissodactylus schmitti* | CH708-H09 | BOLD:ADE2628 | PINMX153-18 | MW544291 | Panteon beach, Oaxaca, Mexico |
| *Fabia subquadrata* | EU329156 | BOLD:ADM0052 | GBCMD18816-19 | EU329156 | ND |
| **Holothuriophilus trapeziformis* | CH670-A02 | BOLD:ADE9974 | PINMX002-16 | MW544424 | Agua Blanca Beach, Oaxaca, Mexico |
| **Holothuriophilus trapeziformis* | CH670-A04 | BOLD:ADE9974 | PINMX004-16 | MW544295 | Agua Blanca Beach, Oaxaca, Mexico |
| **Holothuriophilus trapeziformis* | CH670-A05 | BOLD:ADE9974 | PINMX005-16 | MW544389 | El Tejon Bay, Oaxaca, Mexico |
| **Holothuriophilus trapeziformis* | CH670-A06 | BOLD:ADE9974 | PINMX006-16 | MW544419 | El Tejon Bay, Oaxaca, Mexico |
| **Holothuriophilus trapeziformis* | CH708-H08 | BOLD:ADE9974 | PINMX152-18 | MW544434 | La Tijera beach, Oaxaca, Mexico |
| **Holothuriophilus trapeziformis* | CH736-A2 | BOLD:ADE9974 | PINMX158-18 | MW544439 | Los Pinitos Bay, Sinaloa, Mexico |
| **Holothuriophilus trapeziformis* | CH736-A3 | BOLD:ADE9974 | PINMX159-18 | MW544442 | Los Pinitos Bay, Sinaloa, Mexico |
| **Holothuriophilus trapeziformis* | CH736-A4 | BOLD:ADE9974 | PINMX160-18 | MW544394 | Los Pinitos Bay, Sinaloa, Mexico |
| **Holothuriophilus trapeziformis* | CH736-A5 | BOLD:ADE9974 | PINMX161-18 | MW544323 | Los Pinitos Bay, Sinaloa, Mexico |
| **Holothuriophilus trapeziformis* | CH736-A6 | BOLD:ADE9974 | PINMX162-18 | MW544407 | Los Pinitos Bay, Sinaloa, Mexico |
| **Holothuriophilus trapeziformis* | CH736-A7 | BOLD:ADE9974 | PINMX163-18 | MW544357 | Los Pinitos Bay, Sinaloa, Mexico |
| **Holothuriophilus trapeziformis* | CH736-A8 | BOLD:ADE9974 | PINMX164-18 | MW544383 | Agua Blanca beach, Oaxaca, Mexico |
| **Holothuriophilus trapeziformis* | CH736-A9 | BOLD:ADE9974 | PINMX165-18 | MW544406 | Panteon beach, Oaxaca, Mexico |
| **Holothuriophilus trapeziformis* | CH736-A10 | BOLD:ADE9974 | PINMX166-18 | MW544338 | Coral beach, Oaxaca, Mexico |
| **Holothuriophilus trapeziformis* | CH736-A12 | BOLD:ADE9974 | PINMX168-18 | MW544404 | Nudista beach, Guerrero Mexico |
| **Holothuriophilus trapeziformis* | CH736-B5 | BOLD:ADE9974 | PINMX173-18 | MW544413 | Caleta de Chon, Guerrero, Mexico |
| **Holothuriophilus trapeziformis* | CH736-B6 | BOLD:ADE9974 | PINMX174-18 | MW544428 | Caleta de Chon, Guerrero, Mexico |
| **Holothuriophilus trapeziformis* | CH736-B7 | BOLD:ADE9974 | PINMX175-18 | MW544408 | Caleta de Chon, Guerrero, Mexico |
| **Holothuriophilus trapeziformis* | CH736-B8 | BOLD:ADE9974 | PINMX176-18 | MW544331 | Zacatoso, Guerrero, Mexico |
| **Holothuriophilus trapeziformis* | CH736-B9 | BOLD:ADE9974 | PINMX177-18 | MW544313 | Zacatoso, Guerrero, Mexico |
| **Holothuriophilus trapeziformis* | CH736-B10 | BOLD:ADE9974 | PINMX178-18 | MW544409 | Zacatoso, Guerrero, Mexico |
| **Holothuriophilus trapeziformis* | CH736-G5 | BOLD:ADE9974 | PINMX233-18 | MW544388 | Camaron beach, Oaxaca, Mexico |
| **Holothuriophilus trapeziformis* | CH736-G6 | BOLD:ADE9974 | PINMX234-18 | MW544441 | Camaron beach, Oaxaca, Mexico |
| **Holothuriophilus trapeziformis* | CH736-G7 | BOLD:ADE9974 | PINMX235-18 | MW544417 | San Agustin Bay, Oaxaca, Mexico |
| **Holothuriophilus trapeziformis* | CH736-G8 | BOLD:ADE9974 | PINMX236-18 | MW544292 | San Agustin Bay, Oaxaca, Mexico |
| **Holothuriophilus trapeziformis* | CH736-G10 | BOLD:ADE9974 | PINMX238-18 | MW544363 | San Agustin Bay, Oaxaca, Mexico |
| **Holothuriophilus trapeziformis* | CH736-G11 | BOLD:ADE9974 | PINMX239-18 | MW544392 | San Agustin Bay, Oaxaca, Mexico |
| **Holothuriophilus trapeziformis* | CH736-G12 | BOLD:ADE9974 | PINMX240-18 | MW544393 | San Agustin Bay, Oaxaca, Mexico |
| **Holothuriophilus trapeziformis* | CH736-H1 | BOLD:ADE9974 | PINMX241-18 | MW544361 | San Agustin Bay, Oaxaca, Mexico |
| **Holothuriophilus trapeziformis* | CH736-H2 | BOLD:ADE9974 | PINMX242-18 | MW544395 | San Agustin Bay, Oaxaca, Mexico |
| **Holothuriophilus trapeziformis* | CH736-H3 | BOLD:ADE9974 | PINMX243-18 | MW544322 | Estacahuite beach, Oaxaca, Mexico |
| **Holothuriophilus trapeziformis* | CH736-H4 | BOLD:ADE9974 | PINMX244-18 | MW544370 | Estacahuite beach, Oaxaca, Mexico |
| **Holothuriophilus trapeziformis* | CH736-H5 | BOLD:ADE9974 | PINMX245-18 | MW544296 | Estacahuite beach, Oaxaca, Mexico |
| **Holothuriophilus trapeziformis* | CH736-H7 | BOLD:ADE9974 | PINMX247-18 | MW544307 | Estacahuite beach, Oaxaca, Mexico |
| **Holothuriophilus trapeziformis* | CH736-H8 | BOLD:ADE9974 | PINMX248-18 | MW544348 | Estacahuite beach, Oaxaca, Mexico |
| **Holothuriophilus trapeziformis* | CH736-H10 | BOLD:ADE9974 | PINMX250-18 | MW544330 | Estacahuite beach, Oaxaca, Mexico |
| **Holothuriophilus trapeziformis* | CH736-H11 | BOLD:ADE9974 | PINMX251-18 | MW544386 | Estacahuite beach, Oaxaca, Mexico |
| *Holothuriophilus pacificus* | ZSMA20111423 | BOLD:ABV9743 | CFAD062-11 | MW544379 | Playa Caballito, Chile |
| *Pinnixa* | NJGS-45 | BOLD:AAN6250 | NJCGS300-10 | HQ966502 | Ocean, 10m deep, Canada |
| *Pinnixa* | HLC-24106 | BOLD:AAG9840 | DQCS021-08 | MG319650 | Canada |
| *Pinnixa faba* | BIOUG01750-F03 | BOLD:ACL8418 | CAISN348-12 | MW544426 | planktonic marine zone using 250um net. Canada |
| *Pinnixa faba* | BIOUG03170-E04 | BOLD:ACL8418 | CAISN432-13 | MW544384 | planktonic marine zone using 250um net. Canada |
| *Pinnixa faba* | BIOUG03173-A02 | BOLD:ACL8418 | CAISN667-13 | MW544425 | planktonic marine zone using 250um net. Canada |
| *Pinnixa faba* | CIB369 | BOLD:ACL8418 | CAPBS001-16 | MW544306 | Departure Bay, Canada |
| *Pinnixa faba* | CIB370 | BOLD:ACL8418 | CAPBS002-16 | MW544356 | Departure Bay, Canada |
| *Pinnixa franciscana* | MBI-SCCWRP-00053 | BOLD:AAO4832 | CMBIA053-10 | HQ941718 | outer harbor, USA |
| *Pinnixa franciscana* | MBI-SCCWRP-00459 | BOLD:AAO4832 | CMBIA523-12 | MW544301 | Station SWOO 32, USA |
| *Pinnixa franciscana* | MBI-SCCWRP-00460 | BOLD:AAO4832 | CMBIA524-12 | MW544328 | Station SWOO 32, USA |
| *Pinnixa franciscana* | BIOUG01212-F03 | BOLD:AAO4832 | ZPC159-14 | MW544399 | Newport Pier, USA |
| *Pinnixa franciscana* | BIOUG01218 C01 Zoea | BOLD:AAO4832 | ZPC217-14 | MW544345 | Newport Pier, USA |
| *Pinnixa franciscana* | BIOUG01218 E07 Zoea | BOLD:AAO4832 | ZPC247-14 | MW544367 | Off Newport Aquatic Center, USA |
| *Pinnixa franciscana* | CCDB 24622 G06 | BOLD:AAO4832 | ZPC650-18 | MW544369 | Balboa at Coral, USA |
| *Pinnixa franciscana* | CCDB 24622 G07 | BOLD:AAO4832 | ZPC651-18 | MW544312 | Balboa at Coral, USA |
| *Pinnixa franciscana* | BIOUG01207-H01 | BOLD:AAO4832 | ZPC085-13 | MW544355 | Newport Pier, USA |
| *Pinnixa franciscana* | BIOUG01226-C01 | BOLD:AAO4832 | ZPC312-15 | MW544435 | Newport Pier, USA |
| *Pinnixa longipes* | MBI-SCCWRP-00055 | BOLD:AAO4831 | CMBIA055-10 | HQ941720 | Station A3, USA |
| *Pinnixa occidentalis* | MBI-SCCWRP-00137 | BOLD:AAY3673 | CMBIA137-11 | MW544315 | Station 0B, USA |
| *Pinnixa tubicola* | CIB179 | BOLD:ACK9533 | CACIB110-16 | KX039763 | Port Hardy Bay, Canada |
| *Pinnixa tubicola* | BIOUG01207-B11 | BOLD:ACK9533 | ZPC023-13 | MW544440 | Off NAC, USA |
| *Pinnixa valdiviensis* | ZSMA20111419 | BOLD:AAZ5354 | CFAD058-11 | MW544402 | Playa Trincao, Chile |
| *Pinnixa valdiviensis* | ZSMA20111420 | BOLD:AAZ5354 | CFAD059-11 | MW544418 | Playa Inio, Chile |
| *Pinnixa valdiviensis* | ZSMA20111422 | BOLD:AAZ5354 | CFAD061-11 | MW544376 | Playa Trincao, Chile |
| *Pinnaxodes* | ZSMA20111439 | BOLD:ABV8132 | CFAD085-11 | MW544427 | Comau Fjord/Punta Cruesa, Chile |
| Pinnotheridae | BIOUG01717-G02 | BOLD:ACK9533 | CAISN1404-13 | MW544414 | planktonic marine zone using 250um net. Canada |
| Pinnotheridae | BIOUG01749-A09 | BOLD:ACL8730 | CAISN199-12 | MW544366 | planktonic marine zone using 250um net. Canada |
| Pinnotheridae | BIOUG03170-E05 | BOLD:ACL8419 | CAISN433-13 | MW544303 | planktonic marine zone using 250um net. Canada |
| Pinnotheridae | BIOUG01226-H08 | BOLD:ACL8419 | ZPC379-15 | MW544293 | Off Newport Aquatic Center, Upper Newport Bay, USA |
| **Pinnotheres* sp. | CH708-H12 | ND | PINMX156-18 | N/A | Cacaluta Bay, Oaxaca, Mexico |
| **Pinnotheres* sp. | CH708-H10 | ND | PINMX154-18 | N/A | San Agustin beach, Oaxaca, Mexico |
| **Pinnotheres* sp. | CH736-D1 | BOLD:ADY0051 | PINMX193-18 | MW544358 | La Blanca, Oaxaca, Mexico |
